# Chronic Kidney Disease Progression and Transition Probabilities in a Large Preventive Cohort in Colombia

**DOI:** 10.1155/2021/8866446

**Published:** 2021-03-31

**Authors:** Jasmin I. Vesga, Edilberto Cepeda, Campo E. Pardo, Sergio Paez, Ricardo Sanchez, Rafael M. Sanabria

**Affiliations:** ^1^Therapy Services-Colombia, Bogotá, Colombia; ^2^Statistics Department, Universidad Nacional de Colombia, Bogotá, Colombia; ^3^School of Medicine, Universidad Nacional de Colombia, Bogotá, Colombia; ^4^Renal Therapy Services-Latin America, Bogotá, Colombia

## Abstract

**Background:**

Variability in chronic kidney disease (CKD) progression is a well-known phenomenon that underlines the importance of characterizing the said outcome in specific populations. Our objectives were to evaluate changes in the estimated glomerular filtration rate (eGFR) over time and determine the frequency of dialysis admission and factors associated with this outcome, to estimate the rate of program's loss-to-follow-up and the probability of transition between CKD stages over time.

**Methods:**

The study type was an observational analytic retrospective cohort in patients treated in a CKD prevention program in Bogota, Colombia, between January 1, 2009, and December 31, 2013, with follow-up until December 31, 2018. Adult participants of 18 years of age or older with diagnosed CKD stages G3 or G4 were enrolled into a prevention program. For each patient, the rate of progression of CKD in ml/min/1.73 m^2^/year was estimated using the ordinary least-squares method. Dialysis initiation and program's loss-to-follow-up rates were calculated. Heat maps were used to present probabilities of transitioning between various CKD stages over time. Survival model with competing risks was used to evaluate factors associated with dialysis initiation.

**Results:**

A total of 2752 patients met inclusion criteria and contributed with 14133 patient-years of follow-up and 200 dialysis initiation events, which represents a rate of 1.4 events per 100 patient-years (95% CI 1.2 to 1.6). The median change of the eGFR for the entire cohort was −0.47 ml/min/1.73 m^2^ per year, and in the diabetic population, it was −1.55 ml/min/1.73 m^2^ per year. The program's loss-to-follow-up rate was 2.6 events per 100 patient-years (95% CI 2.3 to 2.9). Probabilities of CKD stage transitions are presented in heat maps. Female sex, older age, baseline eGFR, and serum albumin were associated with lower risk of dialysis initiation while CKD etiology diabetes, cardiovascular disease history, systolic blood pressure, blood urea nitrogen, and LDL cholesterol were associated with a higher likelihood of dialysis initiation.

**Conclusions:**

A CKD secondary prevention program's key indicator is reported here, such as dialysis initiation, progression rate, and program drop-out; CKD progression appears to be correlated with diabetic status and timing of referral into the preventive program.

## 1. Introduction

Chronic kidney disease (CKD) is a syndrome that today has important implications for the health of populations and the economic sustainability of health systems around the world [1]. In 2012, the KDIGO expert consensus updated and redefined the clinical guidelines for the evaluation, management, and treatment of patients with CKD, which today serve as a guide for the care of these patients [2]. Epidemiologically in CKD, two population groups can be identified: one in the general population with more representation of older individuals, with few comorbidities and a very low rate of progression; and another in patients referred to CKD specialized services, with a greater proportion of acquired or hereditary nephropathies, with a greater impairment of renal function and a higher rate of progression to renal failure [3]; these two populations frequently intersect in CKD preventive programs.

The literature shows a significant variability in CKD progression rates, with substantial proportions of populations that progress less than −1 ml/min/year, other populations with progression rates at around −4 ml/min year, and groups that evolve quickly and reach −10 ml/ml/year [4–7]. Linear regression methods have been used to estimate the progression of CKD based on multiple measurements of the estimated glomerular filtration rate from serum creatinine [8]. It should also be noted that staging based on these estimates has a high degree of variability between patients and intra-patient, with an acceptable longitudinal performance [9, 10].

Based on this class of methods, a population follow-up study with 10-year follow-up in 3047 patients in Norway reported a rate of progression of CKD of −1.03 ml/min/1.73 m^2^ per year, with an incidence cumulative renal failure in the 10 years of 0.04 (95% CI 0.03–0.06) [11]. A study by Keith et al. showed that the rate of initiation to kidney replacement therapy was lower than mortality in this population [12].

There is scarcity of data about the transition between CKD stages over time; however, extensive research has been done regarding models that predict the progression of this disease [13], as well as variables associated with fast or slow progression [14, 15].

In Colombia, a prevention program for CKD was implemented to identify patients early with a high risk of progression and with uncontrolled risk factors such as hypertension out of goals, poor glycemic control, and proteinuria and to offer multidisciplinary care interventions (nephrologist, nurse, dietitian, social work, and psychology), to improve control of their disease and outcomes [16].

For context, Renal Therapy Services (RTS) in Colombia is a renal care provider network caring for approximately 9500 patients in CKD prevention programs. Compensar is a Colombian Health Maintenance Organization that provides and insures healthcare for 1.5 million patients. More than a decade ago, RTS and Compensar established a CKD prevention program, with the goal of early detection of CKD patients in stages G3 or G4 and intervening to reduce the rate of progression and ensure a gradual, planned transition to initiate kidney replacement therapy, when needed.

The study aimed to evaluate changes in the estimated glomerular filtration rate (eGFR) over time, as well as to determine the frequency of dialysis initiation and factors associated with this outcome, to determine the rate of program's loss-to-follow-up, and the probability of transition between CKD stages over time.

## 2. Materials and Methods

### 2.1. Type of Study

The study type was observational, analytic, historical cohort.

### 2.2. Population

All patients admitted to the CKD prevention program of Renal Therapy Services (RTS) and Compensar between January 1, 2009, and December 31, 2013, with CKD stages G3 or G4, older than 18 years, were assessed for inclusion; the end of the follow-up duration was December 31, 2018. To be included in the study cohort, patients had to have at least two eGFR <60 ml/min/1.73 m^2^ and a diagnosis of CKD by a nephrologist; follow-up duration was a minimum of 5 years. The presence of metastatic disease was considered an exclusion criterion. Patients entered the cohort on the date of first estimate of the GFR. The main outcomes of the study were initiation of dialysis, transitions between CKD stages, and loss-to-follow-up. Censoring occurred for the following events: referral to primary care, change of provider/insurance, death, suspension from the program, palliative care, and kidney transplant.

At least four estimates of the glomerular filtration rate with the Chronic Kidney Disease Epidemiology Collaboration (CKD-EPI) equation [17] were required to estimate CKD progression rate; this minimum number of eGFR measurements was established in order to estimate the linear trend of eGFR over time as it has been used in other studies [8, 10]; this trend allows establishing whether there is progression, remission, or regression of CKD [18].

### 2.3. CKD Secondary Prevention Program

RTS CKD prevention program was an integrated approach between the primary and secondary levels of care [16]. In its initial phase, an educational program was implemented to ensure that primary care physicians identify and screen patients with a need for referral to the secondary level. Comprehensive assessments and interventions occur quarterly for patients with CKD stage G3 and monthly for stages G4 and G5.

The interventions encompassed laboratory monitoring, nephrology consultation, nutritional evaluation and treatment, and comprehensive CKD education by a nurse and social worker to decrease CKD progression and prepare the patient for eventual kidney replacement therapy.

Patients were admitted to the program when they had at least two eGFRs measuring <60 ml/min/1.73 m^2^ and clinical diagnoses of CKD.

### 2.4. Procedures for Variable Measurement and Gathering of Information

The variables measured in the study were extracted from the RTS electronic medical records system (Versia®), which systematically records the measurements that are part of standard clinical care of these patients on a quarterly or monthly basis according to the standard of care protocols. A project information engineer and a quality information assurance person were responsible for building and validating the research database. Data corresponding to variables measured in this study were transferred from Versia® to an Excel file and then exported to a Stata format for statistical analysis, using STATA 14® (Stata Corp LLC, College Station, Texas) and R version 3.6.2 program.

### 2.5. Statistical Analysis

For the descriptive component, the following variables were captured: age, sex, ethnicity, education level, CKD etiology, history of cardiovascular disease, glomerular filtration rate estimated by the CKD-EPI formula [19], CKD stage, body mass index, systolic and diastolic blood pressure, serum albumin, hemoglobin, glycosylated hemoglobin in diabetics, blood urea nitrogen, uric acid, 24-hour proteinuria, LDL cholesterol, and use of angiotensin-converting enzyme inhibitors (ACEIs) and angiotensin II receptor blockers (ARBs). In addition, the arrival at CKD stage G5 and the initiation of kidney replacement therapy (KRT) with dialysis were recorded as a dichotomous variable. For descriptive analyses, percentages, means (Standard deviation, SD), or medians (interquartile range, IQR) were used according to the type of variable analyzed.

The follow-up duration was divided into 6-month periods. Permanence counts were made at the end of each period. The counts were presented in figures as proportion of CKD stage for every period. For each patient, the CKD progression rate was estimated in ml/min/1.73 m^2^/year, as the slope of the individual linear regression line of eGFR over follow-up using the ordinary least-squares regression; as used in other studies [14] a negative slope indicates CKD progression. Each patient contributed at least four follow-up measurements of eGFR; the mean, SD, median, and 25th and 75th percentiles were reported.

The rate of dialysis initiation and the program's loss-to-follow-up were estimated as incidence rates and reported as events per patient-year, with its respective 95% CI. Additionally, the transition probabilities between the different CKD stages over six-month periods were estimated and this information was presented using heat maps; each cell contains the probability expressed as percentage (using as a numerator the number of patients passing from one stage in rows to a columns stage, and as denominator the total of patients for each row). Probabilities of transition were estimated for the total CKD population, diabetic, hypertensive, and glomerulonephritis.

We followed the Fine & Gray approach to fitting a survival model with competing risks. No data imputation procedure was performed, as the models were built on patients with complete information. The survival model incorporating competing risk of death, kidney transplant, and palliative care included the following variables: time to start dialysis, age, sex, education level, cardiovascular disease history, CKD etiology, body mass index, systolic and diastolic blood pressure, estimated glomerular filtration rate, serum albumin, hemoglobin, blood urea nitrogen, uric acid, and LDL cholesterol.

The study was approved by the RTS Ethics Research Committee, approved on January 20, 2015, that exempted the use of informed consent as this study does not contain identifiable information and is an observational study.

## 3. Results

**Figure 1:**
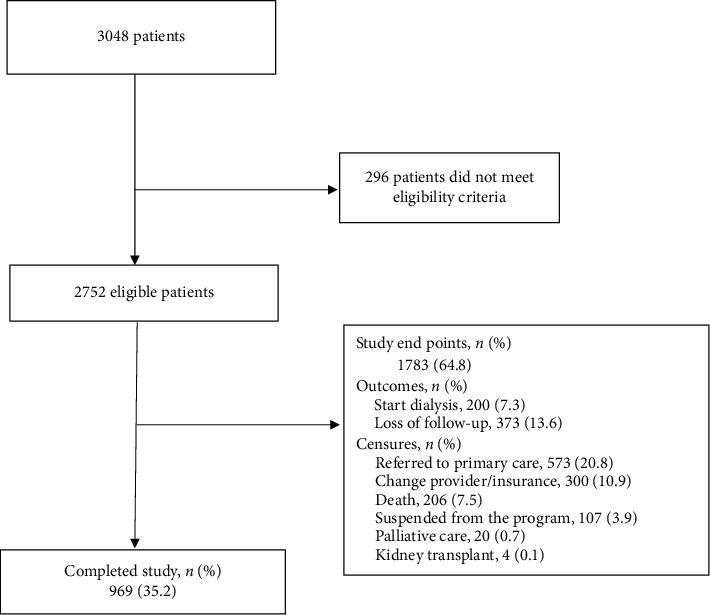
Flow chart of patients in the study. The diagram shows the flow of patients in the study. Of the 3048 originally recruited patients, 296 did not meet the eligibility criteria. 969 patients finished the study without presenting an outcome or censure events.

A total of 2752 patients were included for analysis. The mean baseline eGFR was 43.2 ml/min/1.73 m^2^ (SD = 9.8); CKD etiology consisted of 60.3% (*n* = 1660) hypertensive nephropathy and 18.4% (*n* = 506) diabetes mellitus; details of baseline demographic and clinical characteristics are presented in Table 1. At the end of the follow-up period, 7.3% (*n* = 200) of patients started dialysis, 13.6% (*n* = 373) had been lost to follow-up, 7.5% (*n* = 206) died, and 20.8% (*n* = 573) were referred to ordinary primary consult; patient's flow within the study is presented in Figure 1.

**Figure 2:**
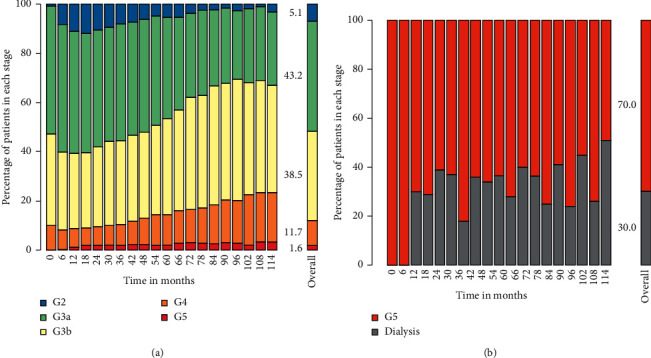
Permanence counts by CKD stages. Each bar's height represents the proportion of patients by CKD stage at the end of each 6-month period. On the right is a subset of patients who achieve CKD stage G5 or dialysis. The overall bar represents the total proportion of all counts during follow-up, expressed as a percentage.

Upon admission into the study cohort, most of patients were in CKD stage G3; and throughout the study, 43.2% of patients' eGFR counts observed in periods of 6 months belong to stage G 3A (see Figure 2). In addition, 67.5% of patients' eGFR counts in CKD stage G5 observed in periods of 6 months remained without requiring dialysis initiation (see Figure 2).

The median number of days in CKD stage G5 without dialysis was 278.5; see Table 2.

All patients contributed 14,133 patient-years of follow-up; there were a total of 200 events of dialysis initiation, which represents an incidence rate of 1.4 events per 100 patients-years (95% CI 1.2 to 1.6); this rate was 3.6 per 100 patient-years for diabetics; otherwise, the program's loss-to-follow-up rate was 2.6 events per 100 patient-years (95% CI 2.3 to 2.9); characteristics of the patients who initiated dialysis are presented in Table 2.

**Figure 3:**
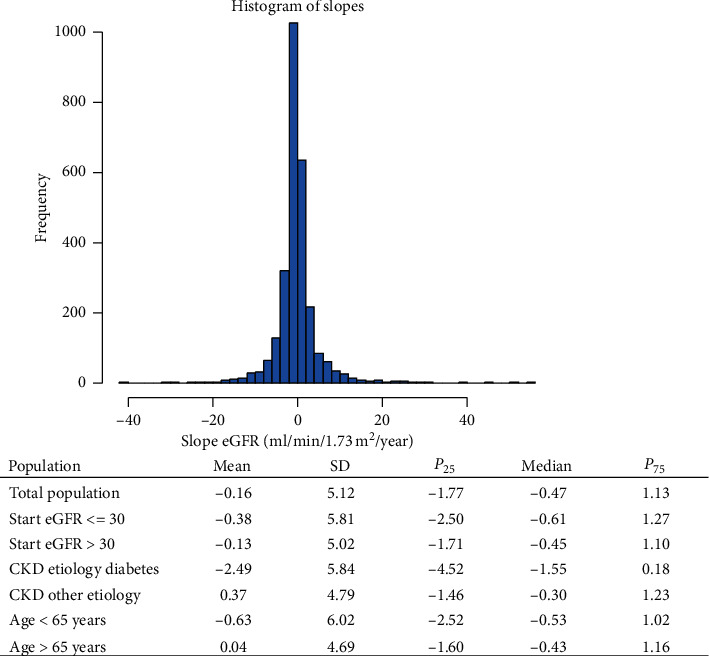
CKD progression. Histogram representing frequency distribution of eGFR slopes in the full sample.

The median change of the eGFR of enrolled patients was −0.47 ml/min/1.73 m^2^ per year, and in diabetic population it was −1.55 ml/min/1.73 m^2^ per year. In the 75th percentile, there is a proportion of patients maintaining their eGFR, some even with improvement; see Figure 3.

**Figure 4:**
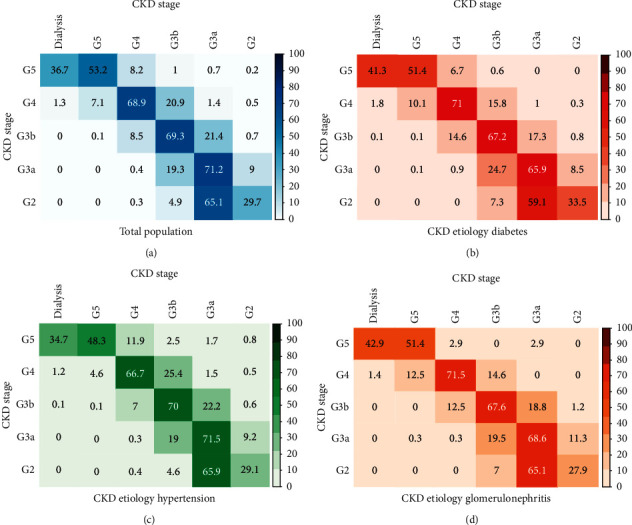
Transition probabilities between CKD stages by etiology. Transitions between CKD stages in 6-month intervals for the total population, diabetic, hypertensive, and glomerulonephritis populations are presented, e.g., on top left panel (total population) a patient in stage G5, after six months, has a 36.7% probability of starting dialysis, 53.2% of staying in stage G5 without dialysis, 8.2% of returning to stage G4, and so on.

Probabilities of transition between CKD stages in six month intervals are shown in Figure 4, including the entire population, diabetic, hypertensive, and glomerulonephritis patient population. Observation of these heat maps demonstrates that the patients tend to remain in their own CKD stage and that the probability of transitioning to a higher stage of renal function deterioration increases in patients whose CKD etiology is diabetes and glomerulonephritis.

Multivariable analyses were conducted to assess the effect of demographic, clinical, and laboratory variables on the risk of dialysis initiation adjusting for the competing risks death, kidney transplant, and palliative care. Female sex, older age, baseline eGFR, and serum albumin were associated with lower risk of dialysis initiation while CKD etiology diabetes, cardiovascular disease history, systolic blood pressure, blood urea nitrogen, and LDL cholesterol were associated with a higher likelihood of dialysis initiation; see Table 3.

## 4. Discussion

The nephrology community has walked a journey in identifying, understanding progression, and estimating the prognosis of patients with CKD [2, 19]. This study conducted in a large number of patients with CKD stages G3, G4, and G5 reports a median eGFR progression of approximately −0.5 mil/min/1.73 m^2^ per year showing that the patients of this program behave in a stable manner in terms of maintaining eGFR. This can be seen indirectly with the low rate of dialysis initiation, close to 1.5 events per 100 patient-years. These results may be related to the fact that a significant proportion of patients entered the CKD prevention program relatively early in their disease course at CKD stage G3, with a median eGFR at baseline of about 43 ml/min/1.73 m^2^.

The speed of progression is lower than other studies reported in the literature [10, 20] which may also be related to the systematic and multidisciplinary intervention of the program designed to decrease CKD progression. Additionally, in these types of prevention programs, so-called fast progressors and slow progressors coexist in a manner where a summary measure of progression may not accurately represent the entire population, with results influenced by a greater proportion of slow progressors within the program [21].

On the other hand, other studies report different progression characteristics and probabilities [22]. This variability in the estimations of CKD progression could represent differences in the studied populations. An important finding of this study is the estimation in likelihood of change between CKD stages, both for the total population and for CKD secondary to diabetes, hypertension, and glomerulonephritis, providing valuable information for physicians, policy makers, and health economists; this data, besides predictive modeling [13], can delineate clinical pathways and patterns of health resources utilization. The variability of measurements (creatinine and eGFR) [23, 24] may affect the probabilities of change between CKD stages, especially for measures that are close to the upper or lower limits between the different stages.

A salient characteristic of this population is the low rate of patients being lost to follow-up, which suggests high adherence to this prevention program where most patients remained in CKD stages G3 and G4. The emphasis on adherence to the program is one of the pillars of its success, since the drop-out of preventive interventions is one of the barriers in health systems. Better results can be achieved with educational and awareness programs for CKD patients. On the other hand, high adherence may reflect additional characteristics that were not measured, as greater tendency to self-care, have healthy life habits, and have adequate health insurance.

This study strengths include data originating from an established CKD prevention program in a large population of patients, standardized interventions/assessments, and a minimum follow-up period of 5 years.

Our study found several factors, similar to prior literature, associated with a lower risk of start dialysis, such as older age, female sex, serum albumin, and baseline estimated glomerular filtration rate [6, 11, 13]. Likewise, we observed that a history of cardiovascular disease, CKD etiology diabetes, systolic blood pressure level [25], blood urea nitrogen, and LDL cholesterol were associated with a higher risk of dialysis initiation, also comparable to that seen in other reports [25–28]. In contrast to several other studies, several factors were not associated with this outcome, including body mass index and uric acid [29].

Study limitations include lack of data on micro-albuminuria, proteinuria, or serum bicarbonate during the study which lead to the inability to analyze these important markers of progression.

## 5. Conclusions

Key indicators of a CKD secondary prevention program's performance are reported in this study, such as rate of initiation of dialysis therapy, drop-out of the program, rate of CKD progression, and the probability of change between stages. CKD progression was associated with diabetic status and the time of entry into the preventive program. These kinds of prevention programs can be a suitable tool to improve patient and health system outcomes.

## Figures and Tables

**Table 1:** Baseline characteristics of the study population.

Baseline characteristics	*N* = 2752	
Female sex, *n* (%)	1429	51.9
Age, mean (SD)	70.4	11.3

Ethnicity, *n* (%)		
Indigenous	4	0.2
African-American	3	0.1
Mestizo	2745	99.7

Education level, *n* (%)		
Elementary or none	787	28.6
High school	1781	64.7
University degree	284	6.7

CKD etiology, *n* (%)		
Hypertension	1660	60.3
Diabetes	506	18.4
Urinary tract obstruction	122	4.4
Glomerulonephritis	84	3.1
Polycystic kidney disease	34	1.2
Other	222	8.1
Unknown	124	4.5

History of cardiovascular disease, *n* (%)	122	4.4
Glomerular filtration rate ml/min/1.73 m^2^, mean (SD)	43.2	9.8
CKD stage G3, *n* (%)	2436	88.5
CKD stage G4, *n* (%)	316	11.5
Body mass index, mean (SD) kg/m^2^	26.8	4.3
Systolic blood pressure mmHg, mean (SD)	134.4	20.8
Diastolic blood pressure mmHg, mean (SD)	74.4	11.7
Serum albumin g/dL mean (SD)	4.2	0.3
Hemoglobin g/dL mean (SD)	14.3	1.9
Blood urea nitrogen mg/dL, median (IQR)	25	21, 33
Uric acid mg/dL mean (SD)	6.7	1.6
Glycosylated hemoglobin %, median (IQR) ^*∗*^	7	6, 8
LDL cholesterol mg/dL, median (IQR)	114	91, 1
24-hours proteinuria, g/L, median (IQR) ^*∗∗*^	0.1	0.1, 0.4
Use of ACEIs and ARBs, *n* (%)	450	16.4
Follow-up time, years, median (IQR)	5.2	3.1, 7.1

^*∗*^Data available only in diabetes patients.  ^*∗∗*^Data available in 1795 patients. CKD: chronic kidney disease; ACEIs: angiotensin-converting enzyme inhibitors; ARBs: angiotensin ii receptor blockers; LDL: low-density lipoprotein.

**Table 2:** Outcomes and baseline characteristics of the population dialysis initiation.

Outcomes	Dialysis initiation incidence rate (per 100 person-years) 95% CI

Total population	1.4 (1.2–1.6)
CKD etiology diabetes	3.6 (2.9–4.5)
CKD other etiology	0.9 (0.7–1.1)
Sex: Male	1.7 (1.5–2.1)
Sex: Female	1.1 (0.9–1.3)

Days in stage 5 without dialysis, median (IQR)¶	278.5 (111, 617)

Dialysis initiation characteristics	*N* = 200
Age, mean (SD) years	61.8 (12.1)
Sexo: Male	121 (60.5)
Diabetes history, *n* (%)	92 (46.0)
Hypertension history, *n* (%)	64 (32.5)
Cardiovascular disease history, *n* (%)	7 (3.5)
Body mass index mean (SD)	27.1 (4.5)
eGFR on beginning, mean (SD) ml/min/1.73 m^2^	10.5 (3.1)
Hemodialysis, *n* (%)	114 (57.0)
Dialysis peritoneal, *n* (%)	86 (43.0)
Planned dialysis start, *n* (%)	89 (44.5)

Dialysis access, *n* (%)	
Arteriovenous fistula	19 (9.5)
Arteriovenous graft	1 (0.5)
Vascular catheter	94 (47.0)
Peritoneal catheter	86 (43.0)

Hospitalization requirement, *n* (%)ǂ	90 (45.0)
Albumin g/dL mean (SD)	3.6 (0.5)
Hemoglobin g/dL mean (SD)	10.6 (1.6)
Calcium mg/mL mean (SD)	8.4 (0.8)
Phosphorous mg/mL mean (SD)	4.9 (1.3)
PTHi pg/mL, median (IQR)	230.5 (135, 367.1)
Iron mcg/dL, median (IQR)	52.2 (34.8, 69.5)
Ferritin ng/mL, median (IQR)	291.6 (128.2, 548.8)

ǂAdmission 3 days before or 3 days after the dialysis start. ¶Days in stage 5 without dialysis.

**Table 3:** Fine & Gray subdistribution hazard competing risk regression model for the dialysis initiation outcome.

Characteristics	SHR	*p* value	95% CI	
Age, 18 to 64 years	Reference	—	—	—
>=65 years	0.30	0.001	0.21	0.42

Sex: Male	Reference	—	—	—
Female	0.38	0.001	0.26	0.55

Education level: elementary or none	Reference	—	—	—
High school	1.12	0.537	0.78	1.62
University degree	0.81	0.554	0.41	1.62

Cardiovascular history: yes	2.89	0.022	1.17	7.17

CKD etiology: others	Reference	—	—	—
Diabetes	3.00	0.001	1.61	5.61
Hypertension	0.96	0.911	0.51	1.82
Glomerulonephritis	1.59	0.253	0.72	3.55
Urinary tract obstructive	0.94	0.897	0.37	2.41

Body mass index, kg/m^2^	1.01	0.621	0.97	1.05
Systolic blood pressure, mmHg	1.01	0.002	1.01	1.02
Diastolic blood pressure, mmHg	1.00	0.673	0.99	1.02
Glomerular filtration rate ml/min/1.73 m^2^	0.94	0.001	0.93	0.96
Serum albumin, g/dL	0.23	0.001	0.17	0.30
Hemoglobin, g/dL	0.94	0.162	0.87	1.02
Blood urea nitrogen, mg/dL	1.01	0.041	1.00	1.03
Uric acid, mg/dL	1.02	0.717	0.93	1.11
LDL-cholesterol, mg/dL	1.00	0.008	1.00	1.01

SHR: subdistribution hazard; CKD: chronic kidney disease; LDL: low-density lipoprotein.

## Data Availability

The full protocol and database for the study can be acquired from the principal investigator (jasmin_vesga@baxter.com).
